# Supplementary material for: Red light-transmittance bagging promotes carotenoid accumulation of grapefruit during ripening
Source: Commun Biol. 2022 Apr 4;5:303. doi: 10.1038/s42003-022-03270-7 (PMC8980019; doi:10.1038/s42003-022-03270-7)
Supplement: Supplementary file 2 — Supplementary Information [file 42003_2022_3270_MOESM2_ESM.pdf]

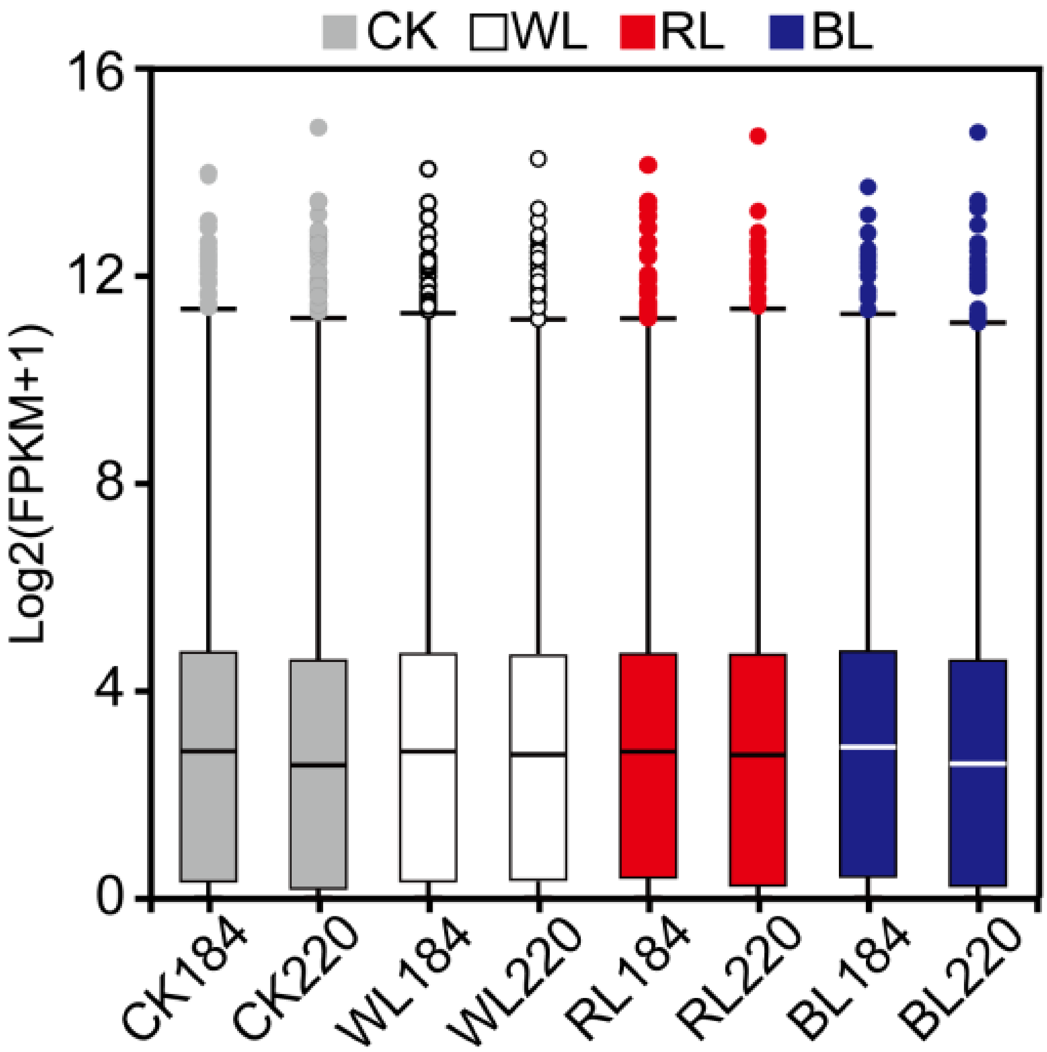


**Supplementary Figure 1.** Box-plot representation of distribution of gene expression level for RNA-seq during grapefruit ripening. RPKM, Reads Per Kilobase transcriptome per Million mapped reads; CK: control group; WL: white light treatment; RL: red light treatment; BL: blue light treatment; DAB184 and 220 represent 184 and 220 days after blossom, respectively. The grey, white, red and blue Box-plots represent the samples treated with control, white light, red light and blue light, respectively.

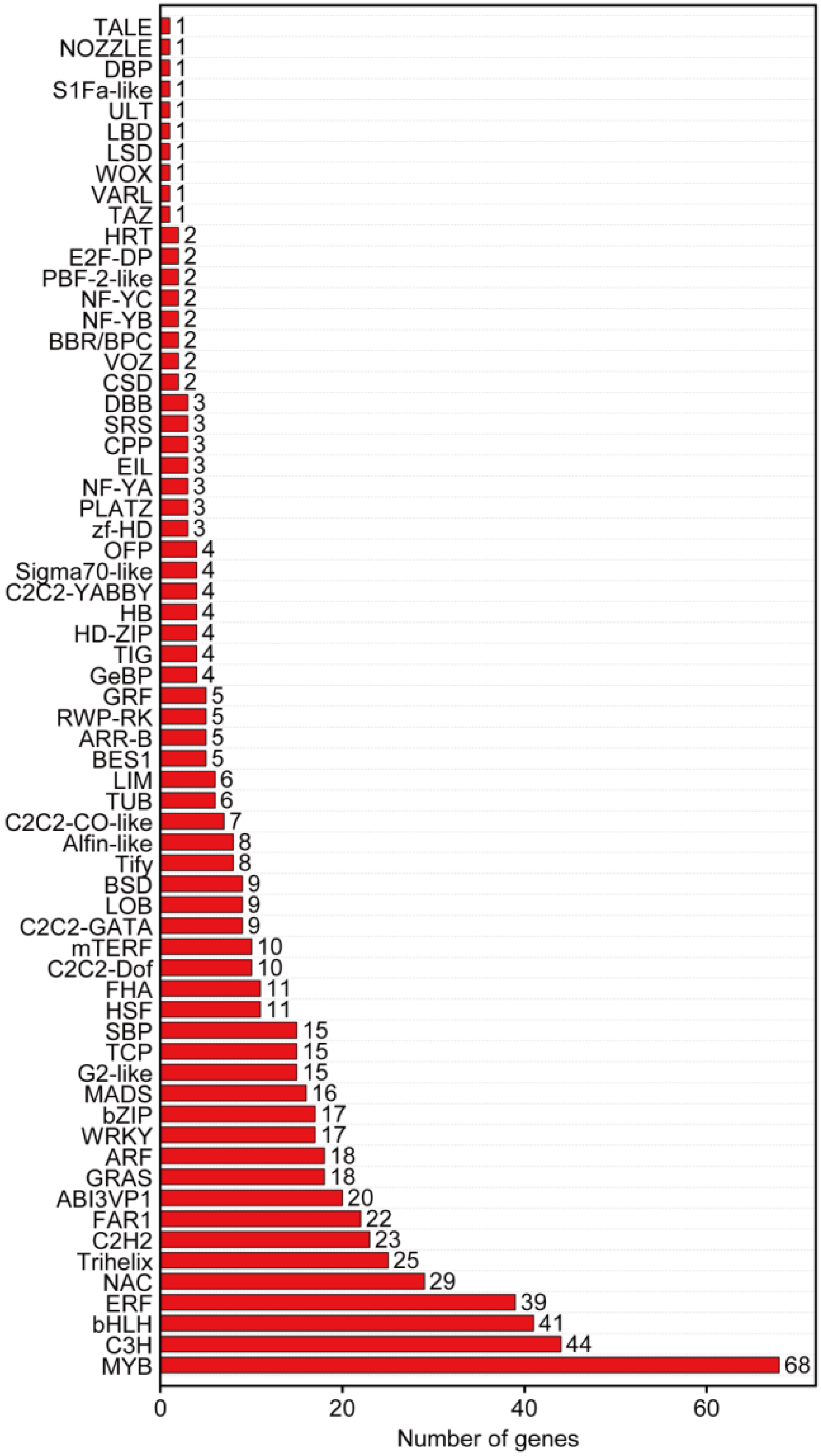
TALE 1
NOZZLE 1
DBP 1
S1Fa-like 1
ULT 1
LBD 1
LSD 1
WOX 1
VARL 1
TAZ 1
HRT 2
E2F-DP 2
PBF-2-like 2
NF-YC 2
NF-YB 2
BBR/BPC 2
VOZ 2
CSD 2
DBB 3
SRS 3
CPP 3
EIL 3
NF-YA 3
PLATZ 3
zf-HD 3
OFP 4
Sigma70-like 4
C2C2-YABBY 4
HB 4
HD-ZIP 4
TIG 4
GeBP 4
GRF 5
RWP-RK 5
ARR-B 5
BES1 5
LIM 6
TUB 6
C2C2-CO-like 7
Alfin-like 8
Tify 8
BSD 9
LOB 9
C2C2-GATA 9
mTERF 10
C2C2-Dof 10
FHA 11
HSF 11
SBP 15
TCP 15
G2-like 15
MADS 16
bZIP 17
WRKY 17
ARF 18
GRAS 18
ABI3VP1 20
FAR1 22
C2H2 23
Trihelix 25
NAC 29
ERF 39
bHLH 41
C3H 44
MYB 68
0
20
40
60
Number of genes

**Supplementary Figure 2.** Enrichment analysis of TFs in carotenoid co-expression modules.

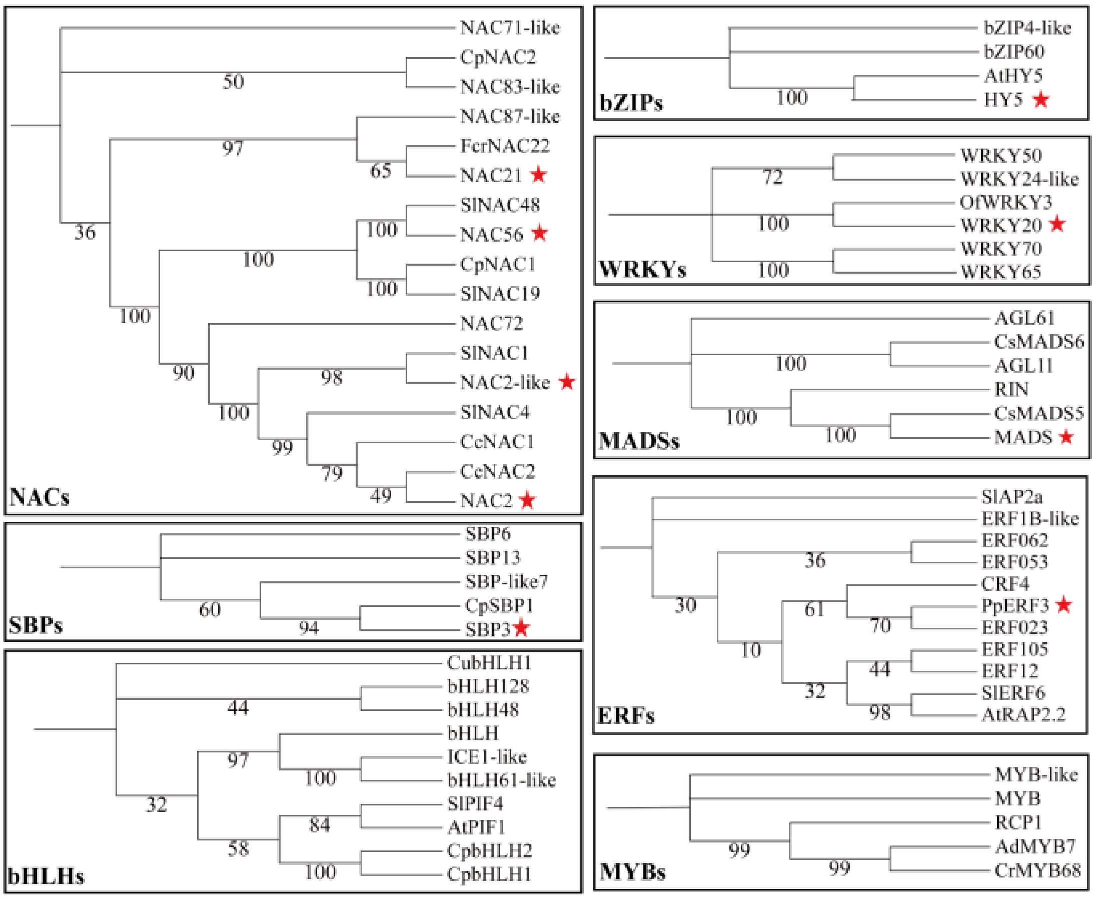


**Supplementary Figure 3.** Phylogenetic analysis of candidate NACs, SBPs, bHLHs, bZIPs, WRKYs, MADSs, ERFs and MYBs transcription factor. Accession number for transcription factors in phylogenetic tree were listed in **Supplementary Table 6**.

a b

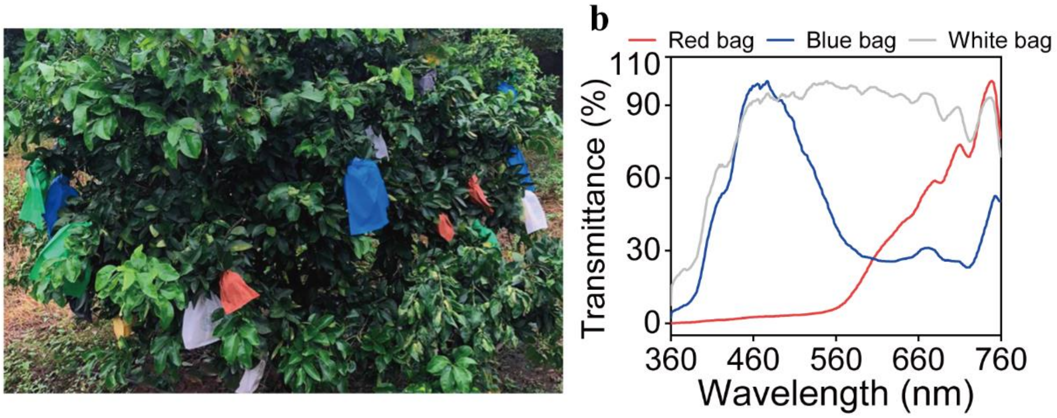


c

| | CK | WL | RL | BL |
|---|---|---|---|---|
| Light intensity ($\mu$mol $m^{-2}$ $s^{-1}$) | 250.21±5.35 | 183.72±3.44 | 145.39±3.11 | 123.78±2.16 |
| Temperature ($^{O}$C) | 19.61±0.58 | 20.37±0.74 | 20.63±0.56 | 20.6±0.41 |
| Relative humidity (%) | 55.67±2.11 | 53.57±1.94 | 55.63±1.87 | 54.97±1.69 |
| $CO_2$ concentration (%) | 0.20±0.03 | 0.23±0.04 | 0.22±0.03 | 0.23±0.04 |
| $O_2$ concentration (%) | 20.10±1.01 | 20.21±0.86 | 20.20±0.74 | 20.21±0.62 |

**Supplementary Figure 4.** Grapefruit-bagging treatment in the field. **a** Grapefruit bagged with light-transmitting bags in the field. **b** Light-transmitting property of bags used for bagging. **c** The micro-climate condition of different light-transmitting bags. Data are expressed as means ± standard deviation of triplicate samples (n = 3). CK: control group; WL: white-light treatment; RL: red-light treatment; BL: blue-light treatment. All parameters were measured at 10:00 AM in October 20, 2020 (a sunny day).

**Supplementary Table 1.** Retention time and maximum absorption peaks of carotenoids standards.

| NO. | Carotenoids | Rt (min) | $\lambda_{max}$(nm) observed | | | $\lambda_{max}$(nm) literature[a] | | |
|---|---|---|---|---|---|---|---|---|
| | | | Peak I | Peak II | Peak III | Peak I | Peak II | Peak III |
| 1 | 9-cis-violaxanthin | 18.15 | 412 | 436 | 462.7 | 411 | 434 | 463 |
| 2 | phytofluene | 32.41 | 329 | 347.1 | 364 | 331 | 347 | 364 |
| 3 | ζ-carotene | 39.53 | 380 | 398.5 | 422.7 | 380 | 400 | 426 |
| 4 | β-carotene | 46.62 | 426 | 451.8 | 478.5 | 426 | 451 | 473 |
| 5 | lycopene | 49.00 | 446.9 | 474.8 | / | 446 | 473 | 503 |

[a] Characterization of Pinalate, a novel Citrus sinensis mutant with a fruit-specific alteration that results in yellow pigmentation and decreased ABA content.

**Supplementary Table 2.** Total RNA quality of RNA-seq samples during grapefruit ripening.

| Sample | Concentration(ng/μL) | Volume(μL) | Total content(μg) | RIN/RQN | 28S/18S | Results |
|---|---|---|---|---|---|---|
| CK184_1 | 645 | 30 | 19.35 | 9.1 | 1.3 | A |
| CK184_2 | 470 | 30 | 14.1 | 8.9 | 1.2 | A |
| CK184_3 | 305 | 30 | 9.15 | 9.2 | 1.3 | A |
| CK220_1 | 420 | 30 | 12.6 | 8.6 | 1.1 | A |
| CK220_2 | 520 | 30 | 15.6 | 8.6 | 1.2 | A |
| CK220_3 | 347 | 30 | 10.41 | 8.4 | 1.2 | A |
| WL184_1 | 233 | 30 | 6.99 | 8.4 | 1.2 | A |
| WL184_2 | 219 | 30 | 6.57 | 8.4 | 1.1 | A |
| WL184_3 | 440 | 30 | 13.2 | 8 | 1.2 | A |
| WL220_1 | 350 | 30 | 10.5 | 8.5 | 1.3 | A |
| WL220_2 | 505 | 30 | 15.15 | 8.8 | 1.3 | A |
| WL220_3 | 365 | 30 | 10.95 | 8.7 | 1.4 | A |
| RL184_1 | 620 | 30 | 18.6 | 8.9 | 1.2 | A |
| RL184_2 | 735 | 30 | 22.05 | 9 | 1.3 | A |
| RL184_3 | 500 | 30 | 15 | 9 | 1.5 | A |
| RL220_1 | 219 | 30 | 6.57 | 7.8 | 1 | A |

| | | | | | | |
|---|---|---|---|---|---|---|
| RL220_2 | 310 | 30 | 9.3 | 8.4 | 1.1 | A |
| RL220_3 | 156 | 30 | 4.68 | 8.4 | 1 | A |
| BL184_1 | 545 | 30 | 16.35 | 8.7 | 1.2 | A |
| BL184_2 | 640 | 30 | 19.2 | 8.2 | 1.2 | A |
| BL184_3 | 600 | 30 | 18 | 9.2 | 1.1 | A |
| BL220_1 | 320 | 30 | 9.6 | 8.3 | 1 | A |
| BL220_2 | 290 | 30 | 8.7 | 8.6 | 1.1 | A |
| BL220_3 | 480 | 30 | 14.4 | 8.5 | 1.1 | A |

Note: CK: control group; WL: white light treatment; RL: red light treatment; BL: blue light treatment; 184, 220 represent 184, 220 days after blossom, respectively. "A" refers to the samples which quality meet the requirements of library construction and sequencing.

**Supplementary Table 3.** Throughput and quality of RNA-Seq data in grapefruit fruit.

| Sample | Total Raw Reads (M) | Total Clean Reads (M) | Total Clean Bases (Gb) | Clean Reads Q20 (%) | Clean Reads Q30 (%) | Clean Reads Ratio (%) |
|---|---|---|---|---|---|---|
| CK184_1 | 48.93 | 43.94 | 6.59 | 96.88 | 90.8 | 89.79 |
| CK184_2 | 48.93 | 45.43 | 6.81 | 97.01 | 91.14 | 92.83 |
| CK184_3 | 48.93 | 45.27 | 6.79 | 96.82 | 90.62 | 92.52 |
| CK220_1 | 48.93 | 45.12 | 6.77 | 96.95 | 90.98 | 92.21 |
| CK220_2 | 48.93 | 44.3 | 6.65 | 96.96 | 91 | 90.54 |
| CK220_3 | 48.93 | 44.88 | 6.73 | 96.97 | 91.02 | 91.71 |
| WL184_1 | 48.93 | 45.28 | 6.79 | 96.76 | 90.44 | 92.54 |
| WL184_2 | 48.93 | 45.29 | 6.79 | 96.88 | 90.78 | 92.56 |
| WL184_3 | 48.93 | 44.97 | 6.75 | 96.89 | 90.81 | 91.9 |
| WL220_1 | 48.93 | 45.46 | 6.82 | 96.82 | 90.63 | 92.9 |
| WL220_2 | 48.93 | 44.92 | 6.74 | 96.87 | 90.76 | 91.79 |
| WL220_3 | 48.96 | 42.58 | 6.39 | 96.72 | 90.21 | 86.98 |
| RL184_1 | 50.68 | 45.07 | 6.76 | 97.03 | 91.13 | 88.93 |
| RL184_2 | 50.68 | 44.29 | 6.64 | 96.98 | 90.98 | 87.38 |
| RL184_3 | 50.68 | 44.97 | 6.75 | 96.6 | 89.91 | 88.74 |
| RL220_1 | 50.68 | 44.09 | 6.61 | 96.66 | 90.08 | 87 |

| | | | | | | |
|---|---|---|---|---|---|---|
| RL220_2 | 50.14 | 43.09 | 6.46 | 96.72 | 90.21 | 85.95 |
| RL220_3 | 50.64 | 43.99 | 6.6 | 96.45 | 89.46 | 86.87 |
| BL184_1 | 50.68 | 44.8 | 6.72 | 96.88 | 90.67 | 88.39 |
| BL184_2 | 47.19 | 44.65 | 6.7 | 96.65 | 90.04 | 94.62 |
| BL184_3 | 47.19 | 44.45 | 6.67 | 96.59 | 89.86 | 94.21 |
| BL220_1 | 47.19 | 44.92 | 6.74 | 96.62 | 89.97 | 95.19 |
| BL220_2 | 47.19 | 44.93 | 6.74 | 96.48 | 89.59 | 95.21 |
| BL220_3 | 47.19 | 44.67 | 6.7 | 96.62 | 89.95 | 94.68 |

Note: CK: control group; WL: white light treatment; RL: red light treatment; BL: blue light treatment; 184, 220 represent 184, 220 days after blossom, respectively. Q20: the rate of bases which quality is greater than 20; Q30: the rate of bases which quality is greater than 30.

**Supplementary Table 4.** Summary of reference genome and gene sequence mapping of grapefruit during ripening after RNA-seq.

| Sample | Total Clean Reads (M) | Reference genome | | Reference gene sequence | |
|---|---|---|---|---|---|
| | | Total Mapping (%) | Uniquely Mapping (%) | Total Mapping (%) | Uniquely Mapping (%) |
| CK184_1 | 43.94 | 86.03 | 82.82 | 75.21 | 71.41 |
| CK184_2 | 45.43 | 87.19 | 83.86 | 78.7 | 74.46 |
| CK184_3 | 45.27 | 86.73 | 83.46 | 77.16 | 73.25 |
| CK220_1 | 45.12 | 87.58 | 83.74 | 77.37 | 72.91 |
| CK220_2 | 44.3 | 87.15 | 83.35 | 76.64 | 72.23 |
| CK220_3 | 44.88 | 87.25 | 83.38 | 76.18 | 71.51 |
| WL184_1 | 45.28 | 86.73 | 83.49 | 76.15 | 72.36 |
| WL184_2 | 45.29 | 87.29 | 83.99 | 77.11 | 73.13 |
| WL184_3 | 44.97 | 87.08 | 83.8 | 76.35 | 72.47 |
| WL220_1 | 45.46 | 85.75 | 82.1 | 76.05 | 71.8 |
| WL220_2 | 44.92 | 87.21 | 83.5 | 75.71 | 71.54 |
| WL220_3 | 42.58 | 87.43 | 83.6 | 76.63 | 72.41 |
| RL184_1 | 45.07 | 86.6 | 83.35 | 76.46 | 72.61 |
| RL184_2 | 44.29 | 86.04 | 82.87 | 75.08 | 71.27 |

| | | | | | |
|---|---|---|---|---|---|
| RL184_3 | 44.97 | 86.55 | 83.38 | 76.23 | 72.48 |
| RL220_1 | 44.09 | 86.03 | 82.34 | 75.33 | 71.09 |
| RL220_2 | 43.09 | 86.2 | 82.51 | 75.24 | 71.06 |
| RL220_3 | 43.99 | 86.54 | 82.89 | 75.83 | 71.58 |
| BL184_1 | 44.8 | 86.48 | 83.32 | 75.64 | 71.79 |
| BL184_2 | 44.65 | 87.11 | 83.92 | 77.7 | 73.76 |
| BL184_3 | 44.45 | 86.27 | 83.09 | 75.73 | 71.97 |
| BL220_1 | 44.92 | 87.73 | 84.07 | 77.46 | 73.28 |
| BL220_2 | 44.93 | 86.46 | 82.81 | 76.27 | 72.21 |
| BL220_3 | 44.67 | 87.02 | 83.42 | 76.08 | 71.95 |

Note: CK: control group; WL: white light treatment; RL: red light treatment; BL: blue light treatment; 184, 220 represent 184, 220 days after blossom, respectively.

**Supplementary Table 5.** Expression pattern of differentially expressed genes (DEGs) involved in carotenoid metabolism during grapefruit ripening (from 184 DAB to 220 DAB).

| Gene ID | module | Gene name | Other ID | CK184 | WL184 | RL184 | BL184 | log2 (RL184/CK184) |
|---|---|---|---|---|---|---|---|---|
| 102622692 | blue | ZEP6 | Cs1g04870 | 1.05 | 1.34 | 2.48 | 1.207 | 1.27 |
| Gene ID | module | Gene name | Other ID | CK220 | WL220 | RL220 | BL220 | log2 (RL220/CK220) |
| 102619293 | - | ZEP7 | orange1.1t04051 | 0.00 | 0.000 | 0.02 | 0.00 | 2.40 |
| 102621234 | - | CCD4-1 | Cs8g14150 | 2.057 | 2.75 | 7.92 | 2.873 | 1.83 |
| 102578064 | - | NCED3 | Cs5g14370 | 42.947 | 7.42 | 20.557 | 8.267 | -1.17 |

Note: CK: control group; WL: white light treatment; RL: red light treatment; BL: blue light treatment; 184 and 220 represents 184, 220 days after blossom, respectively.

**Supplementary Table 6.** Gene ID for protein sequence in phylogenic tree.

| Gene name | Gene ID | Gene name | Gene ID |
|---|---|---|---|
| SlNAC48 | NM 001279348 | CrMYB68 | KY612511 |
| FcrNAC22 | XM_006440961 | AdMYB7 | AXP34749.1 |
| CpNAC2 | XP_021899904 | RCP1 | KR053165 |
| SlNAC4 | KC453999 | CubHLH1 | BAU09296.1 |
| SlNAC1 | AY498713 | CpbHLH1 | XP_021890297.1 |

| | | | |
|---|---|---|---|
| CcNAC1 | KC814686 | CpbHLH2 | XP_021907394.1 |
| CcNAC2 | KC814687 | SlPIF4 | Solyc07g043580 |
| SlNAC19 | XM 004236996 | AtPIF1 | At2g20180 |
| CpNAC1 | evm.model.supercontig_1.48 | PpERF3 | Prupe.7G194400 |
| CpSBP1 | evm.model.supercontig_27.40 | AtRAP2.2 | At3g14230 |
| CsMADS6 | MG594037 | SlAP2a | SGN-U314860 |
| CsMADS5 | MK431890 | SlERF6 | JN616265 |
| RIN | NP_001234670.1 | AtHY5 | AT5G11260 |
| OfWRKY3 | KM504380 | | |
