# Supplementary material for: Red light-transmittance bagging promotes carotenoid accumulation of grapefruit during ripening
Source: Commun Biol. 2022 Apr 4;5:303. doi: 10.1038/s42003-022-03270-7 (PMC8980019; doi:10.1038/s42003-022-03270-7)
Supplement: Supplementary file 3 — Description of Additional Supplementary Files [file 42003_2022_3270_MOESM3_ESM.pdf]

Description of Additional Supplementary Files

File name: Supplementary Data 1

Description: Effect of grapefruit bagged with four light-transmitting bags on the TSS content, TA content and CCI of grapefruits during ripening.

File name: Supplementary Data 2

Description: Effects of different light treatments on content of carotenoid, including β-carotene, phytofluene, ζ-carotene, lycopene, and 9-cis-violaxanthin content during grapefruit ripening.

File name: Supplementary Data 3

Description: The number of upregulated and downregulated genes between samples during grapefruit ripening.

File name: Supplementary Data 4

Description: Data for weighted gene co-expression network analysis of grapefruit during ripening under different light-transmittance treatments.

File name: Supplementary Data 5

Description: Expression data of structural genes and transcriptional factors correlated with carotenoid metabolism during grapefruit ripening.

File name: Supplementary Data 6

Description: Expression data of differentially expressed transcription factors in response to RL during grapefruit ripening (from 184 DAB to 220 DAB).

File name: Supplementary Data 7

Description: The co-expression relationship of TFs and structural genes related to carotenoid metabolism in 'blue' module and 'turquoise' module.

File name: Supplementary Data 8

Description: Gene ID for transcription factors and structural genes in the Supplementary Data 7.

File name: Supplementary Data 9

Description: Expression data of 22 important candidate genes involved in the carotenoid metabolism using qRT-PCR and RNA-seq.

File name: Supplementary Data 10

Description: Primer sequences used for real-time quantitative PCR.
